# Plant Sterol-Poor Diet Is Associated with Pro-Inflammatory Lipid Mediators in the Murine Brain

**DOI:** 10.3390/ijms222413207

**Published:** 2021-12-08

**Authors:** Madlen Reinicke, Judith Leyh, Silke Zimmermann, Soroth Chey, Ilijana Begcevic Brkovic, Christin Wassermann, Julia Landmann, Dieter Lütjohann, Berend Isermann, Ingo Bechmann, Uta Ceglarek

**Affiliations:** 1 Institute of Laboratory Medicine, Clinical Chemistry and Molecular Diagnostics, Leipzig University, Liebigstr. 27, 04103 Leipzig, Germany; madlen.reinicke@medizin.uni-leipzig.de (M.R.); silke.zimmermann@medizin.uni-leipzig.de (S.Z.); Soroth.Chey@medizin.uni-leipzig.de (S.C.); ilijana.begcevic@medizin.uni-leipzig.de (I.B.B.); christin.wassermann@gmx.net (C.W.); Berend.Isermann@medizin.uni-leipzig.de (B.I.); 2 Institute of Anatomy, Leipzig University, Liebigstr. 13, 04103 Leipzig, Germany; judith.leyh@medizin.uni-leipzig.de (J.L.); Julia.landmann@medizin.uni-leipzig.de (J.L.); ingo.bechmann@medizin.uni-leipzig.de (I.B.); 3 Institute of Clinical Chemistry and Clinical Pharmacology, University Hospital Bonn, Venusberg-Campus 1, 53127 Bonn, Germany; Dieter.Luetjohann@ukbonn.de

**Keywords:** brain, COX, inflammation, microglia, plant sterols

## Abstract

Plant sterols (PSs) cannot be synthesized in mammals and are exclusively diet-derived. PSs cross the blood-brain barrier and may have anti-neuroinflammatory effects. Obesity is linked to lower intestinal uptake and blood levels of PSs, but its effects in terms of neuroinflammation—if any—remain unknown. We investigated the effect of high-fat diet-induced obesity on PSs in the brain and the effects of the PSs campesterol and β-sitosterol on in vitro microglia activation. Sterols (cholesterol, precursors, PSs) and polyunsaturated fatty acid-derived lipid mediators were measured in the food, blood, liver and brain of C57BL/6J mice. Under a PSs-poor high-fat diet, PSs levels decreased in the blood, liver and brain (>50%). This effect was reversible after 2 weeks upon changing back to a chow diet. Inflammatory thromboxane B2 and prostaglandin D2 were inversely correlated to campesterol and β-sitosterol levels in all brain regions. PSs content was determined post mortem in human cortex samples as well. In vitro, PSs accumulate in lipid rafts isolated from SIM-A9 microglia cell membranes. In summary, PSs levels in the blood, liver and brain were associated directly with PSs food content and inversely with BMI. PSs dampen pro-inflammatory lipid mediators in the brain. The identification of PSs in the human cortex in comparable concentration ranges implies the relevance of our findings for humans.

## 1. Introduction

There is growing evidence that obesity-related cholesterol (CH) and fatty acid-rich diets are among the major determinants of civilization diseases such as diabetes, cardiovascular diseases and neuroinflammatory disorders [1,2,3,4,5]. Plant-derived sterols (plant sterols, PSs) are a group of sterols that are structurally similar to CH with small side chain differences at the C-24 position. In contrast to CH, mammals are not able to synthesize PSs; instead, they are exclusively derived from the food intake of plants, plant oil, seeds or nuts. The habitual diet composition contains a similar amount of CH and PSs. However, compared to CH, PSs concentrations in the plasma are up to 100-fold lower because absorbed PSs are immediately secreted by the ATP-binding cassette (ABC) half-transporters ABCG5 and ABCG8 in the intestine and liver. Daily PSs consumption depends on individual nutritional habits and varies between 178–463 mg/day [6]. There is an increasing interest in PSs because of their CH-lowering properties [7,8] and more recently, anti-inflammatory effects of PSs have been described [9]. Hence, PSs have been explored as a supplement in different experimental and clinical studies to investigate their effect on inflammation-associated biomarkers in the circulation [9,10,11]. The crosstalk between peripheral and cerebral metabolism might open new perspectives in cholesterol management [5].

Animal studies demonstrated that PSs can cross the blood–brain barrier (BBB) via High-Density Lipoprotein (HDL) and/or Apolipoprotein E (ApoE), where they may exert brain cell-type-specific effects [12,13]. Beneficial and adverse effects of PSs on neurodegeneration, central nervous system (CNS) repair and neuroinflammation have been described [1]. The activation of liver-x-receptors (LXR) by PSs was shown to suppress the inflammatory response and promote CNS repair mechanisms [1,14]. PSs are incorporated within lipid rafts (LRs), likely reflecting their closely related structure compared to CH [15,16,17,18]. By lowering the cholesterol content in lipid rafts, PSs inhibit oxidative stress and stimulate the expression of antioxidant molecules [19]. Lipid rafts induced by sitosterol were not as tightly packed as domains formed by other sterols [20], and the dysfunction of lipid rafts disturbs glial function [21,22,23,24,25,26]. Recently, beta-sitosterol was found to inhibit the binding of lipopolysaccharides (LPS) to toll-like receptor 4 in microglia [27]. Microglia mediate immune responses in the central nervous system and the release of inflammatory mediators such as prostaglandins [28]. The interaction of LPS with Toll-like receptor 4 (TLR4)/CD14 triggers intracellular signaling cascades, leading to microglial activation and the transcriptional induction of cyclooxygenase-2 (COX-2), and the subsequent release of prostaglandins. TLR4 signaling, receptor dimerization, requires the ordered membrane microenvironment of LRs. Plant sterols influence membrane fluidity and may influence membrane receptors and channels involved in neuroinflammation [27].

Although the effect of nutritional PS supplementation on brain sterol metabolism has already been investigated, less is known about the influence of obesity-related PS-poor high-fat diet (HFD) on brain sterol metabolism and neuroinflammation. The aim of our study was to investigate the influence of a high-fat diet on brain sterol metabolism and the association of sterol concentrations in brain to pro- and anti-inflammatory signaling lipids and microglial activation. In the brain, little is known about the local distribution of PSs [15,16,17,18,29]. To investigate the local effects, we selected the brain regions of the cortex, cerebellum, hippocampus and hypothalamus, of which the functions are related to cognition in terms of learning, short- and long-term memory, maintaining homeostasis and the planning and execution of movements [30,31,32].

## 2. Results

### 2.1. Sterol Distribution in the Brain, Plasma and Liver with Aging

**Figure 1:**
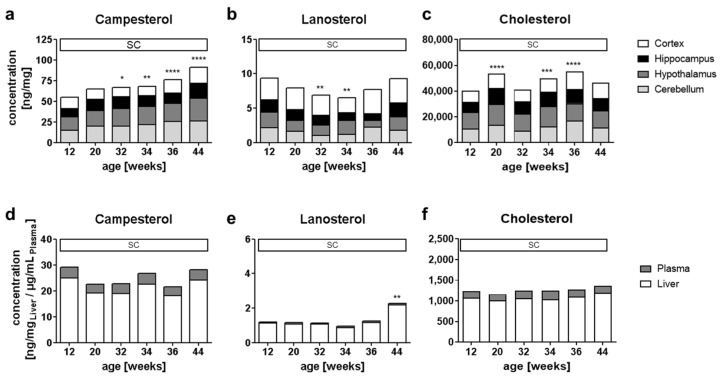
Changes in campesterol (**a**,**d**), lanosterol (**b**,**e**) and cholesterol (**c**,**f**) under a standard chow diet (SD) in the cortex, hippocampus, hypothalamus, cerebellum (**a**–**c**), liver and plasma (**d**–**f**) of male C57BL/6J mice. The height of each bar segment indicates the sterol concentration in each sample specimen; the total bar height corresponds to the total amount; vertical bars reflect the time for the diet type. The statistical effect, according to one-way ANOVA with a post hoc Bonferroni multiple comparisons test, is given in comparison to week 12 with *p* < 0.05 *, *p* < 0.01 **, *p* < 0.001 ***, *p* < 0.0001 ****.

To determine the kinetics and spatial distribution of PSs in the brain, we used targeted lipidomics to measure the PSs campesterol (CA) stigmasterol (ST), sitosterol (SI) and brassicasterol (BR) in the brains of C57BL/6J mice (*n* = six for each time point) fed a standard chow diet (SC; for food composition, see Supplemental Table S1). In Figure 1 the concentrations of CA, LA and CH under SD are shown for the brain regions of the cortex, cerebellum, hippocampus and hypothalamus, as well as the serum and liver. The PS concentrations are summarized in Supplemental Table S2. The following relative distributions of PSs were observed across all timepoints (age 12–44 weeks): 65.1% ± 3.0% CA, 16.7% ± 3.4% ST, 10.7% ± 1.8% SI and 7.4% ± 2.5% BR. The lowest PS amounts were found in the hippocampus and the highest PS amounts were observed within the hypothalamus. Over all four brain regions, CA increased under the chow diet by 167% ± 15% from week 12 to week 44 (*p* < 0.0001), and SI increased by 155% ± 27% (*p* < 0.0001) in the same period (Figure 1a, and Supplemental Figure S1a).

CH and its precursors desmosterol (DE) and lanosterol (LA) were also detected in all four brain regions (Figure 1b,c, Supplemental Figure S1b, Supplemental Table S2). In contrast to PSs, DE and LA displayed a region-specific distribution. The highest amounts of DE and LA were within the hypothalamus and the cortex, whereas the lowest amount of DE was in the cerebellum and LA in the hippocampus. In contrast to PSs DE and CH concentrations did not change with age (Figure 1c, Supplemental Figure S1b). Interestingly, LA showed an age-dependent decrease (*p* = 0.0037) up to the age of 34 weeks, followed by an increase up to an age of 44 weeks in all brain areas (Figure 1b). Overall, the order of sterol concentrations in the brain was CH>>>DE>CA>SI>LA and we calculated a CA/SI ratio of 6:1, which was comparable in all brain regions (Supplemental Table S3). The mean DE/LA ratio was 18:1 in the cortex and cerebellum, but 33:1 in the hippocampus and hypothalamus.

In the liver, only CA and SI were found, with a relative distribution of 75.8% ± 2.6% CA and 24.2% ± 2.6% SI, whereas ST and BR were below the detection limit. In plasma, the relative PS distribution was 69.7% ± 3.8% CA, 27.4% ± 3.6% SI, 2.0% ± 0.4% ST and 0.8% ± 0.3% BR. No age effects were detected in the liver or plasma for PSs until 44 weeks (Figure 1d, Supplemental Figure S1e). Compared to the brain, the concentrations of LA and DE were lower in the liver (30–50% for LA and 60% for DE, Figure 1e, Supplemental Figure S1f, Supplemental Table S2). An age-dependent effect was found only for LA in the liver, showing a significant increase of 100% (Figure 1e), which was not apparent in the plasma. CH and DE concentrations did not change with age (Figure 1f, Supplemental Figure S1f). No significant correlation of PSs with CH or its precursors DE and LA were found (see Supplemental Table S4). Overall, the sterol distribution in the liver and plasma was found to be CH>>>CA>SI>DE=LA, with comparable CA/SI ratios of 3:1 in both matrices. In contrast, the ratio of the cholesterol precursors DE to LA was i3:1 in plasma, but 1:1 in the liver (see Supplemental Table S3).

### 2.2. Influence of Diet on Brain Sterol Concentrations

**Figure 2:**
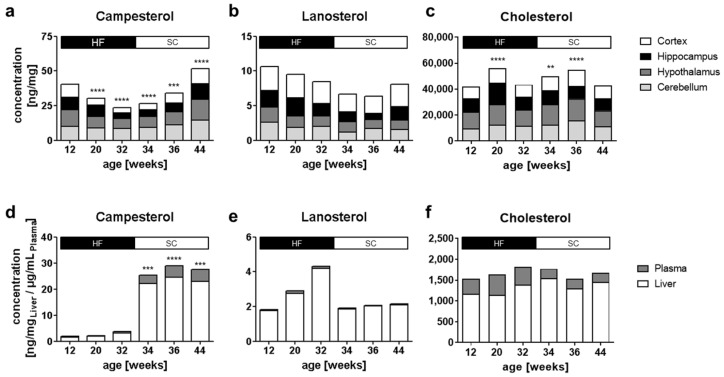
Changes in campesterol (**a**,**d**), lanosterol (**b**,**e**) and cholesterol (**c**,**f**) over the duration of the diet experiment in the cortex, hippocampus, hypothalamus, cerebellum (**a**–**c**), liver and plasma (**d**–**f**) of male C57BL/6J mice. Mice were fed with a high-fat diet (HF) from age 8 to 32 weeks (24 weeks on diet), followed by an SC from 32 to 44 weeks of age (vertical bars reflect the time and type of diet). The height of each bar segment indicates the sterol concentration in each sample specimen; the total bar height corresponds to the total amount; vertical bars reflect the time for the diet type. The statistical effect, according to one-way ANOVA with a post hoc Bonferroni multiple comparisons test, is given in comparison to week 12, with *p* < 0.01 **, *p* < 0.001 ***, *p* < 0.0001 ****.

In the HFD-group, PS concentrations decreased within the experimental time. After 24 weeks of this diet at an age of 32 weeks, mice were 40% heavier (body weight 41.9 ± 3.9 g) compared to mice on the SC diet (body weight 30.0 ± 1.3 g. Details of the HF diet’s food composition and the differences in body weight are given in Supplemental Table S1 and Supplemental Figure S2). PS concentrations differed significantly for all brain regions, as well as for the liver and plasma, starting from diet week 4 (age 12 weeks); see Figure 2d. At the age of 32 weeks, PS concentrations were 50% lower in the HF diet group and the cerebral PS distribution differed significantly compared to the SC diet group (Figure 2a, Supplemental Figure S1c). The order of the relative concentrations of PSs was as follows: CA>ST>BR>SI within the cerebellum and the hypothalamus, whereas in the hippocampus and the cortex the distribution was CA>ST>BR and ST>CA>BR, respectively (Supplemental Table S5). The mean CA/SI ratio was 7.5:1 (Supplemental Table S6). The HF diet-related differences for PSs within the cortex are shown in Supplemental Figure S3a (expressed as fold changes normalized to SC diet). LA was lower in all brain regions after 24 weeks of the HF diet at the age of 32 weeks (Figure 2b). Although DE and CH concentrations showed significant changes, no trend was observed under the HF diet. (Figure 2c, Supplemental Figure S1d). Absolute concentrations and DE/LA ratios were comparable to those of mice fed an SC diet. There was no effect on CH homeostasis in the cortex (Supplemental Figure S3b,c).

Liver tissue showed 86% lower concentrations of CA and SI after 24 weeks of the HF diet, whereas the decrease in the plasma was 74% compared to the SC diet (Figure 1d and Figure 2d, Supplemental Figures S1a,c and S3d,g). ST and BR could not be detected in liver or plasma samples. The CA/SI ratio after 24 weeks of the HF diet was 1:1 in plasma and 6:1 in the liver (Supplemental Table S6). LA and DE concentrations were significantly higher in the liver after the HF diet as opposed to the SC diet (DE ~200%, and LA ~400%; Figure 1b and Figure 2b, Supplemental Figures S1f,h and S3e). Diet did not have an effect on the CH concentration in liver (Figure 1c and Figure 2c, Supplemental Figure S3f). In plasma, DE increased up to 415%, whereas LA was 133% higher after 24 weeks of the HF diet when compared to the SC diet (Figure 1b and Figure 2b, Supplemental Figures S1f,h and S3h). Under the HF diet, the CH concentration increased in plasma up to 232% when compared to the SC diet (Supplemental Figure S3i).

### 2.3. Reversibility of Dietary Effects in Plasma, Liver and Brain

Switching the HF diet group after 24 weeks to the SC diet for an additional 12 weeks (HF-SC diet group) resulted in a 14% weight loss. At the end of the experiment, after 36 weeks, HF-SC mice were 12% heavier than the SC diet group (Supplemental Figure S2). The PS concentrations in the brain increased by 66% (*p* < 0.0001) and the PS distribution was again CA>ST>BR>SI in all brain regions. At the end of the feeding experiment, at an age of 44 weeks, a significant increase in PS concentrations was observed for all PSs: CA, SI, ST and BR (Supplemental Table S5).

In the liver and plasma, the decrease in PS concentrations was completely reversible upon changing mice back to the SC diet (Supplemental Figure S3d,g). The concentrations of CA and SI increased to the levels observed in the SC diet (liver 94% and plasma 115%), and the CA/SI ratio returned to 3:1. DE and LA levels decreased to SC diet levels (DE 117%, and LA 96%). Likewise, plasma CH levels were normalized in the HF-SC diet group. Similar results were found regarding the ratios of CA/CH, SI/CH, DE/CH and LA/CH (Supplemental Table S6).

To determine whether the observed changes in PS concentrations are dependent on the food composition or may be modified by the amount of food intake, we determined PS concentrations in the blood, liver and brain tissues of ob/wt mice (*n* = 5) and ob/ob mice (*n* = 3) at the age of 16 weeks. Due to a mutant and defective leptin synthesis, homozygous ob/ob mice ingest more food than their heterozygous littermates (ob/wt) [33]. Both groups were fed a SC diet (Supplemental Table S1). The body weight of lean ob/+ mice (27.2 ± 0.8 g) was comparable with the body weight of SC-diet-fed C57BL/6J mice at the age of 12 weeks. The body weight of ob/ob mice (52.5 ± 1.2 g) was twice as high as the weight of the ob/+ mice at this time point (*p* < 0.0001). PSs in plasma, the liver and the brain were increased in obese ob/ob mice, whereas LA was significantly decreased and CH was unaffected (Supplemental Figure S4 and Supplemental Table S7). This indicates that the observed PS concentrations in the plasma, liver and brain directly depend on the ingested quantity of PSs in food.

### 2.4. Plant Sterols and COX Activation in the Brain

**Figure 3:**
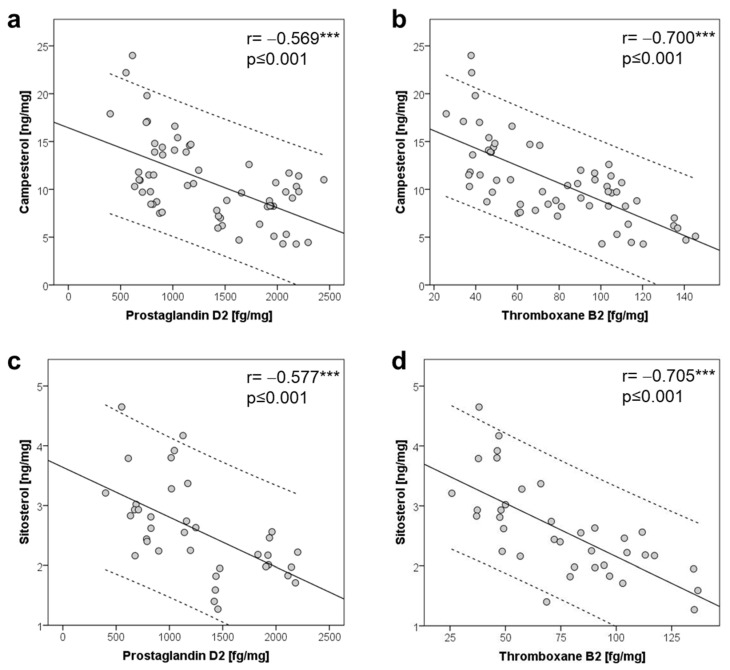
Pearson correlation of campesterol with (**a**) prostaglandin D2 and (**b**) thromboxane B2, and of sitosterol with (**c**) prostaglandin D2 and (**d**) thromboxane B2; 95% confidence interval over all analyzed brain structures (cortex, hippocampus, hypothalamus and cerebellum, *n* = 96) from male C57BL/6J mice aged 12 to 32 weeks and fed a coconut-oil-based high-fat diet, *p* < 0.001 ***.

PS have been proposed to modulate the generation of lipid-derived pro-inflammatory mediators. Hence, we next investigated the correlation of PSs with arachidonic acid (ARA)-derived pro-inflammatory COX-metabolites prostaglandin D2 (PGD2) and thromboxane B2 (TxB2). In all brain regions, regardless of diet, ARA and 19 eicosanoids, including the COX-derived metabolites PGD2 and TxB2, could be quantified. PGD2 was 14–23-fold higher in concentration than TxB2, showing the highest concentration in the cortex. The highest TxB2 concentration was found in the hippocampus (Supplemental Tables S8 and S9, Supplemental Figure S5). A significant indirect correlation was detected for CA and SI, with PGD2 and TxB2 in all brain regions and for all investigated time points, as shown in Figure 3 for mice under the HFD diet (data on SC diet see Supplemental Figure S6). Significant correlations were found for CA with PGD2 (r= −0.569, *p* ≤ 0.001), CA with TxB2 (r= −0.700, *p* ≤ 0.001), SI with PGD2 (r= −0.491, *p* ≤ 0.001) and SI with TxB2 (r= −0.446, *p* ≤ 0.001). Hence, a diet that is poor in PSs or a reduced PS intake can enhance pro-inflammatory COX lipid mediators in the brain, suggesting that a PS-rich diet has anti-inflammatory effects in the brain. Congruently, we observed increased expression of prostaglandin D2 synthase (Ptgds) and thromboxane A synthase-1 (Txbas1) after 4 weeks of the diet, e.g., in the cortex, Supplemental Figure S7. This further supports the hypothesis of a dose-dependent effect of the ingested quantity of PSs.

### 2.5. Plant Sterols and In Vitro Microglia Activation

**Figure 4:**
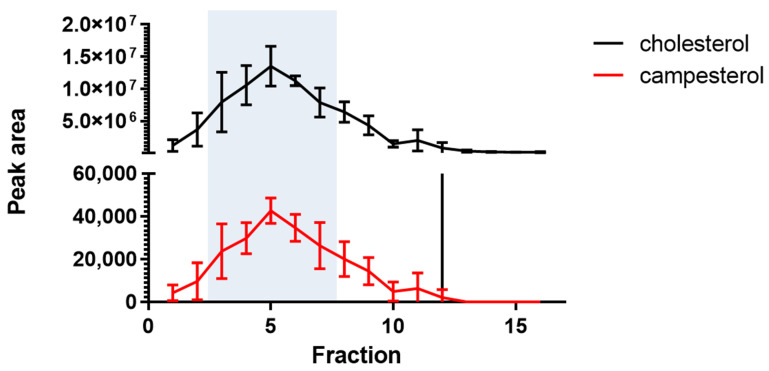
LC-MS/MS analysis of extracted cholesterol (black) and campesterol (red) of isolated lipid raft fractions prepared from murine microglia SIM-A9 using the continuous gradient detergent-free ultra-centrifugation method [34]. The fractions with the highest lipid raft content, determined by Western blot analysis of flotillin (referring to Figure S10), are highlighted in light blue. The sterol content is expressed as the peak area.

The quantification of CA in isolated cell membranes of murine SIM-A9 microglial cells (Figure 4) revealed the highest concentrations in fractions reflecting lipid rafts with a ratio of CA to CH being 1:325 (Supplemental Figure S8). As lipid rafts are thought to contribute to signaling via the assembly of receptorsomes, the accumulation of CA in lipid rafts supports altered receptor activation and signaling in microglia exposed to PSs.

**Figure 5:**
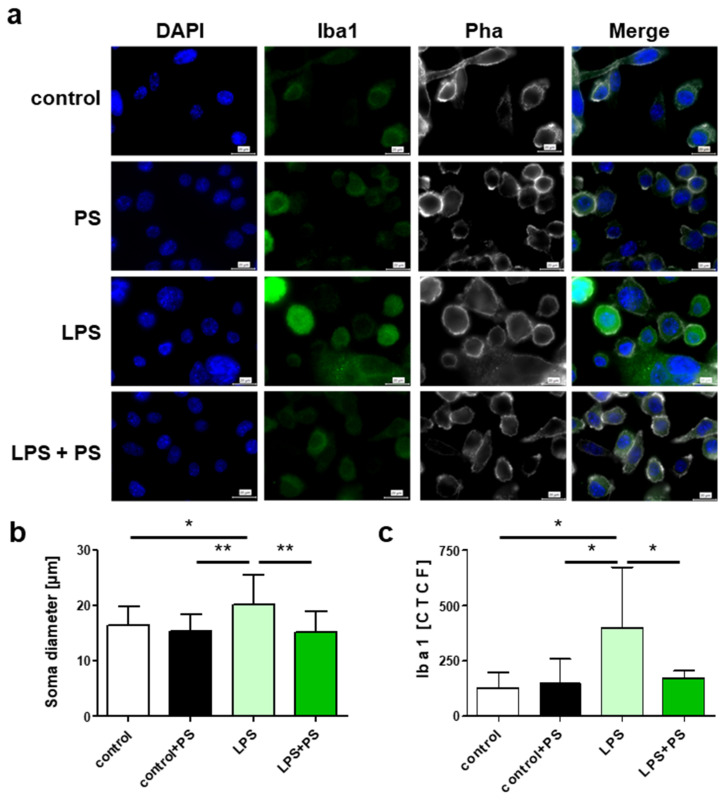
Fluorescence staining of murine microglia SIM-A9 (**a**). 4′,6-diamidino-2-phenylindole (DAPI) (blue) is a nuclear marker and binds strongly to adenine/thymine-rich regions in the DNA. Iba1 (green) is expressed by microglia and circulating macrophages. The F-actin fibers are stained with phalloidin (Pha) (gray), scale bar: 20 µm. (**b**) Soma diameter and (**c**) Iba1 fluorescence intensity expressed as corrected total fluorescence (CTFC). Stimulation with lipopolysaccharide (LPS) without or with the addition of 50 nM phytosterols (PS). Statistics: two-way ANOVA with Tukey’s post hoc multiple comparison test, *p* < 0.05 *, *p* < 0.01 **.

To verify the inverse correlation between PSs and TxB2, as well as PGD2, and to determine whether PSs mediate cell-autonomous effects on microglia cells, we conducted in vitro studies. SIM-A9 microglial cells were stimulated for 12 h using lipopolysaccharide (LPS), tumor necrosis factor alpha (TNFα) or interferon gamma (IFNγ), and two PSs (CA, SI) were added in a physiological concentration of 50 nM. To determine microglia activation, we determined the soma diameter and expression of Iba1 (ionized calcium-binding adapter molecule 1, immunofluorescence), which both reflect the activation status of microglia [35,36]. Compared to control conditions, soma size and Iba1 fluorescence intensity increased significantly in microglial cells stimulated by LPS, TNFα and IFNγ. In the presence of PSs, soma size and Iba1 fluorescence intensity were significantly decreased in all stimulated conditions, matching levels in control conditions without activation (Figure 5 and Supplementary Figure S9).

### 2.6. Plant Sterols in Human Brain

**Figure 6:**
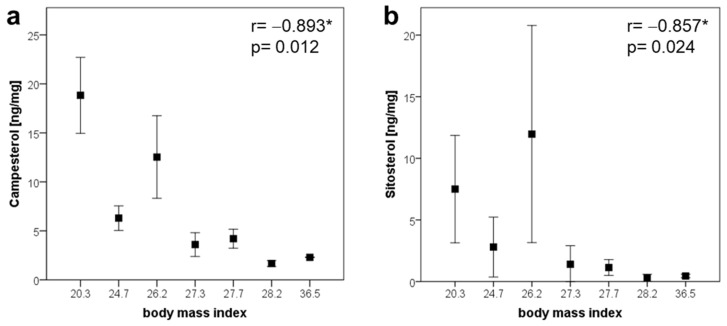
Spearman correlations indicating an association of campesterol (**a**) and sitosterol (**b**) with body mass index in human cortices. Concentration is given as mean ± SD of triplicates. Correlations are significant with *p* < 0.05 *.

To determine whether the above findings may be relevant for humans, we scrutinized the PS concentrations in human brain cortex samples. To this end, we analyzed sterols in the human cortex from seven body donors (age 57–94 years, BMI 20.3–36.5). The absolute concentrations of PSs between 3.7 and 41.9 ng/mg were comparable to those found in mice. Only BR was not detectable. The CA/SI ratio was 2.4:1 and the PSs/CH ratio was 1:1000. Interestingly, we observed a negative association for CA and SI with the BMI (Figure 6). These data indicate that cerebral PS accumulation follows the same pattern as that observed in the circulation, but further analyses have to be carried out [37,38,39,40].

## 3. Discussion

PSs are exclusively derived from the diet, as they cannot be synthesized by mammals. Therefore, their analysis in the blood, liver and brain tissue reflects nutrition-derived lipids and their accumulation in specific body compartments. Here, we showed for the first time that in mice PS concentrations in the plasma, liver and brain directly depend on the ingested quantity of PSs in food. Second, we were able to show a negative correlation between the amount of PSs and the pro-inflammatory COX lipid mediator level in brain. This suggests that food-derived PSs are associated with anti-inflammatory effects in the brain. We observed this effect in both feeding cohorts and a limitation of our study design is that it did not allow us to state that the observed changes in inflammation were related to a diet poor in PSs. Importantly, PSs were identified in the human brain at comparable concentrations and were associated with BMI, which implies the relevance of our findings for humans.

Under the SC diet, containing a total of 220 ± 17 ng/mg PSs, PSs accumulated with age within all brain regions with the following distribution order: CA>>ST>SI>BR. The highest accumulation of CA and SI was found in the cerebellum, as previously described by Vanmierlo et al. [29]. The substitution of SC with a PS-poor HF diet (85 ± 19 ng/mg PSs) resulted in a direct reduction of PS concentrations in the plasma and liver, followed by reduced PS levels in the brain after two weeks. At the end of the HF diet period of 24 weeks, the CA concentration was 75% lower and mice were 40% heavier compared to the SC diet group. These observations correlated directly with the different nutritional composition of 45.5 ± 2.8 ng/mg CA in the SC diet and 11.5 ± 3.3 ng/mg CA in the HF diet but less with body weight. The analysis of the brain obtained from ob/ob mice showed a significant increase of PS concentrations compared to ob/+. Therefore, the accumulation of PSs appears to be related to the amount of PSs by food composition and food intake.

Of note, the HF-diet-dependent effects were reversible upon switching mice to an SC diet. Upon this dietary change, all PSs levels in the plasma, liver and brain significantly increased and the parameter of cholesterol synthesis in the plasma and liver decreased after 14 days of the SC diet. In contrast, the CH concentration did not differ between the different diets, which is in agreement with the findings of Quan et al. [41]. These observations confirmed our findings, that dietary PS content directly affects cholesterol homeostasis in the circulation and brain. The regional differences of the cholesterol synthesis precursor DE and LA may reflect autonomous DE and LA synthesis in the different brain regions. Although we analyzed the composition of the diet, overlapping effects of the HF diet cannot be excluded. This issue, regarding functional mechanisms and relevance, has to be addressed in further feeding experiments.

PSs have been shown to reduce the plasma levels of phospholipase A1 [8]. These effects may be mediated through beneficial alterations in the membrane composition, affecting membrane fluidity and signaling pathways [20,42]. We investigated whether PSs may affect the cellular activation associated with the pro-inflammatory pathway of ARA in the brain. In all brain areas we found an indirect association of PSs with COX-derived eicosanoids PGD2 and TXB2 in both diet groups. Congruently, a previous report described reduced COX expression under PS supplementation [43,44]. COX is the key and rate-limiting enzyme in the conversion of ARA to prostaglandins (PGs), which are lipid metabolites that are involved in several physiological and pathological processes, including inflammation [45]. Our findings support a potential role of nutrition-derived PSs for the regulation of inflammatory processes in the brain. Learning and memory were not affected by HF-induced obesity [46].

As microglia regulate inflammatory processes in the brain and are able to secrete a variety of pro-inflammatory mediators, including eicosanoid [47], we determined the cell-autonomous effects of PSs on COX-dependent lipid-metabolites and inflammatory markers using murine SIM-9 microglia cells. We identified PSs in isolated SIM-A9 cell membranes with a CA/CH ratio of 1:325, which is different from the CA/CH ratio in plasma, suggesting a selective incorporation of PSs in cell membranes. Importantly, the highest CA content was observed in the lipid raft fraction. Upon in vitro stimulation, morphological markers of microglia activation were reduced in the presence of physiological concentrations of CA and SI. In vivo, there is variation in the populations of microglial cells between different brain regions. Densely populated areas were found in the hippocampus and less densely populated areas include the cerebellum [48]. This effect might be in line with the finding of the highest concentration of COX-dependent lipid-metabolites in the hippocampus and the lowest in the cerebellum. Taken together, these findings support a model in which food-derived PSs are taken up into the brain and specifically into microglia cells, dampening inflammatory processes in brain.

Finally, we were able to detect PSs in all of the seven analyzed human cortex samples in comparable concentrations to those found in mice. This indicates that nutritionally derived PSs are physiological components in the human brain and are not only elevated under pathophysiological conditions [49]. Given the fact that PS concentrations influence neuroinflammation, individual nutrition behavior may have a beneficial effect in terms of the decreased development of neuroinflammatory CNS disorders. In summary, here we show that food-derived PSs are taken up by the brain and can be incorporated in the cell membrane. PSs convey anti-inflammatory effects in microglia, potentially through their modification within lipid raft structures or COX-dependent lipid metabolism. These findings have broad implications, as the modification of microglia function through food-derived PSs may convey anti-neuroinflammatory effects and modulate the function of CNS or neurodegenerative diseases.

## 4. Materials and Methods

### 4.1. Experimental Design

Male wild-type C57BL/6J mice were fed either an SC or HF diet for 4, 12 or 24 weeks, followed by further 2, 4 or 12 weeks of an SC diet. Results were verified in a second experiment following the same feeding strategy until 24 weeks and afterwards feeding with an SC diet. The genetic mouse model of ob/ob and ob/+ mice were fed a SC diet for 16 weeks. See Supplementary Materials, Materials and Methods for details of the experimental design. All animal experiments were approved by the local state and university authorities. We performed this study in accordance with the European Commission Recommendations concerning the protection of laboratory animals. The animal experiments were authorized by the local ethics committee of the state of Saxony. Human samples from body donation subjects were collected post mortem, according the written informed consent and authorized by the local ethics committee.

### 4.2. Tissue Preparation for LC-MS/MS

Isolated mouse livers and brain tissues were rinsed in PBS. The brain was carefully dissected to obtain the cortex, cerebellum, hypothalamus and hippocampus. Samples were snap-frozen in liquid nitrogen and stored at −80 °C until further analysis. Liver and brain tissue were homogenized in PBS via sonication and divided into aliquots representing 1 mg of tissue homogenate. Dried samples were then subjected to an analyte-specific extraction procedure [50]. Briefly, dried aliquots of liver and brain substructures (as triplicates) were mixed with 650 μL extraction solvent containing the internal standard (IS). For sterol extraction, *n*-hexane/2-propanol (*i*PrOH) (60:40 *v*/*v*) was used. PUFAs and eicosanoids were extracted in *n*-hexane/*i*PrOH (60:40 *v*/*v*, 0.1% FA). Samples were incubated at 4 °C for 1 h with gentle mixing. After centrifugation at 4 °C, 1000× *g* for 10 min the supernatant was collected and the extraction solvent was evaporated. Dried samples were reconstituted in an LC-MS/MS compatible solvent: sterols in *i*PrOH/methanol (MeOH) (50:50 *v*/*v*), PUFAs and eicosanoids in MeOH/water (62:38 *v*/*v*) and stored at −80 °C until further analysis. EDTA-plasma was also collected and protein precipitation was performed as previously described [51,52].

### 4.3. Quantification of Plasma and Tissue Concentrations via LC-MS/MS

Targeted LC–MS/MS analyses for the quantification of free PSs (CA, SI, ST, BR), LA, DE and CH were performed according to our previously published method [52]. The analysis of ARA and its metabolites PGD2 and TxB2 was performed according to our methods, previously described in [50,51]. For sterol analysis, sample extracts (25 μL) were loaded onto a Chromotlith^®^ SpeedROD RP-18e analytic column (Merck, Darmstadt, Germany) for chromatographic separation using a mobile phase gradient, as described previously [52]. Mass-to-charge ratios of the transitions of interest were monitored in positive ion mode via turbo ion electrospray on an API 4000 triple-quadrupole MS system (SCIEX, Framingham, MA, USA). For PUFA and eicosanoid analysis, an online SPE was implemented into the LC system. Sample extracts (50 μL) were loaded onto a Strata-X extraction column prior to chromatographic separation on a Kinetex^®^ C18 analytic column (Phenomenex Aschaffenburg, Germany), using a mobile phase gradient, as described previously [50,51]. Mass-to-charge ratios of the transitions of interest were monitored in scheduled negative ion mode via turbo ion electrospray on a QTRAP^®^ 5500 MS system (SCIEX, Framingham, MA, USA). The concentration of each analyte in plasma or tissue samples was calculated via interpolation of the absorbed analyte/internal standard peak area ratio into the linear regression line for the calibration curve, which was obtained by plotting peak area ratios versus analyte concentrations and 1/× weighting.

### 4.4. Immunohistochemisty

Staining of immortalized SIM-A9 murine microglial cells was performed using DAPI, Iba1 and Pha. After treatment, cells were washed three times with cold DPBS and then fixed with 4% PFA for 17 min at 37 °C. Fixed cells were washed three times with DPBS and blocked for one hour in blocking solution (DPBS, 3% donkey serum, 0.3% Triton^®^ X 100). Cells were incubated overnight with AIF-1 antibodies (1:250) at 4 °C. The next day, cells were washed with DPBS and incubated with donkey anti-rabbit antibodies (1:200) conjugated with DyLight 488 and Alexa Fluor™ Plus 647 Phalloidin for 2 h at room temperature. Cells were washed with DPBS and mounted with antifade mounting medium with DAPI on cover glasses. Images were taken on a confocal microscope (BZ-X810, KEYENCE DEUTSCHLAND GmbH, Neu-Isenburg, Germany) using a 60× objective lens. For quantification, the corrected total fluorescence (CTFC) was calculated via grayscale conversion of the Fitc channel and measurement of the integrated density of background and single cells, as well as the area of single cells. The equation for CTFC is: integrated density single cell − (area single cell × integrated density mean background integrated density).

### 4.5. Microglia Cell Culture Experiment

Immortalized SIM-A9 murine microglia cells were seeded on coverslips in 24-well plates (40,000 cells per well), in DMEM-F12, supplemented with 5% heat-inactivated horse serum, 10% heat-inactivated fetal bovine serum, 5% penicillin streptomycin and were grown overnight at 37 °C. The cells were treated for 12 h with either 100 ng/mL LPS, 20 ng/mL TNFα or 100 ng/mL IFNγ and with or without a mixture of the phytosterols CA and SI at 50 nM.

### 4.6. Microglia Lipid Raft Isolation and Extraction

Lipid rafts of cultivated SIM-A9 murine microglia cells were isolated using the detergent-free isolation method following the protocol for monolayer-grown cells [34]. The method involves detergent-free isolation, which exploits the flotation of low-density lipid rafts in a continuous iodixanol (OptiPrep™) gradient. Lipids were extracted using the procedure as described previously, with modifications [53]. Briefly, to 150 µL of each fraction, 600 µL of MeOH/*i*PrOH (50:50 *v*/*v*), 600 µL of chloroform and 600 µL of distillated water were added and vortexed for 1 min. The phases were separated via centrifugation at 4 °C and 15,000× *g* for 15 min. The bottom layer (chloroform-lipids phase) was transferred into a new tube. The solvent was evaporated under a nitrogen stream to dryness and the pellet was re-suspended in 100 µL MeOH/*i*PrOH (50:50 *v*/*v*). The solution was ready for mass spectrometric analysis. The purity of the isolated fractions was determined through Western blot analysis; see Supplementary Materials, Materials and Methods.

### 4.7. Quantitative RT-qPCR

The relative quantification of gene expression was determined using the 2−ΔΔCt method (see Supplementary Materials, Materials and Methods).

### 4.8. Data Analysis

All experiments were performed at least in triplicate. For in vivo experiments, samples were collected from 6 animals per treatment group or timepoint (C57BL/6J mice), differently from ob/+ (*n* = 5) and ob/ob (*n* = 3) mice. Results are presented as mean ± SD. All statistical analyses were performed using GraphPad Prism 5 software (GraphPad Software, Inc., San Diego, CA, USA). Statistical significance between two groups was assessed using a two-tailed Student’s *t*-test for unpaired experimental values. For the statistics of microglial cell culture experiments, the Mann–Whitney U test was applied. PSs were correlated with inflammation markers by calculating Pearson correlation coefficients. Multiple group comparisons were performed using one-way ANOVA. A *p*-value of <0.05 was considered statistically significant.

## Data Availability

Collected data are provided in the Supplementary Materials section.

## Supplementary Materials

The following are available online at https://www.mdpi.com/article/10.3390/ijms222413207/s1.
