# Supplementary material for: Plant Sterol-Poor Diet Is Associated with Pro-Inflammatory Lipid Mediators in the Murine Brain
Source: Int J Mol Sci. 2021 Dec 8;22(24):13207. doi: 10.3390/ijms222413207 (PMC8707069; doi:10.3390/ijms222413207)
Supplement: Supplementary file 1 [file ijms-22-13207-s001.zip › 20211207_Supplementary Material_MRe.pdf]

International Journal of
Molecular Sciences

MDPI

Supplementary Materials

Plant Sterol-Poor Diet is Associated with Pro-Inflammatory Lipid Mediators in the Murine Brain

Madlen Reinicke [1], Judith Leyh [2], Silke Zimmermann [1], Soroth Chey [1], Ilijana Begcevic Brkovic [1], Christin Wassermann [1], Julia Landmann [2], Dieter Lütjohann [3], Berend Isermann [1], Ingo Bechmann [2] and Uta Ceglarek [1]*

* Correspondence: uta.ceglarek@medizin.uni-leipzig.de; +49(0) 341 9722200

Materials and methods

Experimental design. All animal experiments were approved by the local state and university authorities. We performed this study in accordance with the European Commission Recommendation 2007/526/CE concerning the protection of laboratory animals. The animal experiments were authorized by the local ethics committee of the state of Saxony (Landesdirektion Sachsen, Leipzig, approval no. TVV 35/12, TVV 01/17 and TVV 41/17). Male wild-type C57BL/6J mice, male ob/ob and ob/+ mice were kept in the local animal facility under a 12 h light and dark cycle with free access to food and water. C57BL/6J mice were fed with a standard chow diet (V1124-300, ssniff Spezialdiaeten, Soest, Germany) and a coconut oil-based cholesterol free high-fat diet (E15772-340, ssniff Spezialdiaeten, Soest, Germany) for up to 44 weeks, detailed illustration can be found in supplemental Figure S10. ob/ob and ob/+ mice were fed with a standard chow diet (V1124-300, ssniff Spezialdiaeten, Soest, Germany) for 16 weeks. Mice were killed under deep anesthesia. EDTA-Plasma was obtained from the right atrial chamber of the heart using a Microvette 200 K3E Capillary Blood Collection System (Sarstedt, Nümbrecht, Germany). After centrifugation for 15 min at 1,000 g, the EDTA-Plasma was snap-frozen in liquid nitrogen and stored at −80 °C until further analysis. Liver and brain were removed, and brain was rinsed in PBS. Cortex, cerebellum, hypothalamus and hippocampus were dissected carefully and immediately. All samples collected were snap-frozen in liquid nitrogen and stored at −80 °C until further analysis.

Chemicals and reagents. The solvents *n*-hexane, 2-propanol and methanol of ultrahigh-performance LC/MS grade were purchased from Biosolve (Valkenswaard, Netherlands). Water was produced in-house with a Barnstead GenPure System from Thermo Scientific (Waltham, MA, USA). Phosphate-buffered saline of liquid, sterile-filtered, suitable-for-cell-culture grade, w/o Ca2+ and w/o Mg2+ (PBS) was obtained from Merck Chemicals (Darmstadt, Germany). Brassicasterol, stigmasterol, desmosterol and cholesterol standards were purchased from Steraloids Inc. (Newport, RI, USA). Campesterol, beta-sitosterol and lanosterol standards were purchased from Sigma-Aldrich (St. Louis, MO, USA). Deuterium-labeled standards as campesterol-d7, sitosterol-d7 were obtained from Medical Isotopes Inc. (Pelham, NH, USA) and cholesterol-d7 was obtained from Cambridge Isotope Laboratories Inc. (Tewksbury, MA, USA). Unlabeled and deuterium-labeled PUFA and eicosanoid standards were purchased from Cayman Chemicals (Ann Arbor, MI, USA); the detailed information can be found in our previously published methods [50, 51].

For fluorescence labeling and cell experiments, the following reagents were used: Immortalized murine microglia cells (SIM-A9) were obtained from ATCC® by LGC Standards GmbH (Wesel, Germany). Dulbecco's modified eagle medium: nutrient mixture F-12 (DMEM/F12), penicillin streptomycin, horse serum, fetal bovine serum, Dulbecco’s phosphate-buffered saline w/o Ca2+ and w/o Mg2+ (DPBS) were purchased from Gibco by Thermo Fisher Scientific (Eugene, OR, USA). Lipopolysaccharide (LPS), paraformaldehyde (PFA) for synthesis and donkey serum were obtained from Sigma-Aldrich (St. Louis, MO, USA). Recombinant carrier-free mouse interferon gamma (IFNγ) and tumor necrosis factor alpha (TNFα) were purchased from Biozol (Eching, Germany). Recombinant monoclonal rabbit AIF-1 antibody was purchased from Novus Biologicals LLC (Centennial, CO, USA). Triton® X 100 and sodium azide were obtained from Carl Roth GmbH + Co. KG (Karlsruhe, Germany). Donkey anti-rabbit IgG (H+L) cross-adsorbed secondary antibody, DyLight 488 and Alexa Fluor™ Plus 647 Phalloidin were purchased from Invitrogen by Thermo Fisher Scientific (Eugene, OR, USA). VECTASHIELD® antifade mounting medium with DAPI was purchased from Vector Laboratories Inc. (Burlingame, CA, USA). Further, for lipid raft isolation and analysis, the following reagents were used: OptiPrep™ from Alere Technologies AS (Oslo, Norway),protease inhibitor cocktail from Promega (Madison, WI, USA),

flotillin-1 (D2V7J) XP® Rabbit mAb and prohibitins (PHB1) Antibody from Cell Signaling Technology Inc. (Danvers, MA, USA), mouse Anti-Calnexin from BD Biosciences (San Jose, CA, USA). BCA™ Protein-Assay, West Pico PLUS chemiluminescent substrate and NuPAGE™ LDS sample buffer from Invitrogen by Thermo Fisher Scientific (Eugene, OR, USA). Goat Anti-Mouse IgG (H + L)-HRP Conjugate, Goat Anti-Rabbit IgG (H + L)-HRP Conjugate, PVDF Transfer Membrane and Protein™ Dual Color Standards from Biorad (Hercules, CA, USA).

Microglia lipid raft isolation and extraction. Murine microglia cells SIM A9 (ATCC® CRL-3265) were grown in DMEM: F12 medium containing 10% heat inactivated fetal calf serum, 5% heat inactivated horse serum and antibiotics penicillin and streptomycin, in a humidified incubator 5% CO2 at 37°C.

The lipid rafts were prepared using the OptiPrep™ (Alere Technologies AS, Norway) continuous gradient detergent-free method of Macdonald and Pike. Briefly, four 150 cm2 plates of confluent SIM A9 cells was washed twice in PBS buffer and once with isolation medium (IM) (250 mM sucrose, 1 mM, CaCl2 and 1 mM MgCl2 and 20 mM Tris-HCl (pH 7.8)). Cells were scraped into IM and pelleted and then lysed in 1 mL of IM containing 1x protease inhibitors cocktail by passage 20 times through a 24-gauge needle and post nuclei supernatants were obtained by low-speed centrifugation (1,000×g, 10 min at 4° C). The remaining pellet was again lysed and centrifuged as aforementioned twice. The three post nuclei supernatants were mixed and made 30% in OptiPrep by addition of 1.5 volume of 50% OptiPrep. Lipid rafts were isolated by centrifugation for 20 h at 31,000 rpm using an SW-40 rotor in a Beckman ultracentrifuge. Fractions of 0.7 mL were collected by aspiration from the meniscus and the distribution of marker proteins was assessed by Western blotting. Total protein in each fraction was determined by BCA™ Protein-Assay (Thermo Fisher Scientific, Eugene, OR, USA).

Western blot analysis. 150 µL of each fraction were precipitated using MeOH/iPrOH/H2O method (40) and the protein pellets were dissolved in 50 µL of 1x NuPAGE™ LDS Sample Buffer (Invitrogen). 20 µL of the protein solution were separated by SDS-PAGE and gels were transferred to PVDF-membrane. The PVDF-membrane was blocked by incubation with 5% non-fat powdered milk. The PVDF strips were incubated for 1 h at room temperature (or overnight at 4°C) with primary antibody (anti-flotillin-1 (1:1000), anti-prohibitins PBH1 (1:5,000) or anti-Calnexin (1:250)), washed, and then incubated with the horseradish peroxidaseconjugated secondary antibody (Goat Anti-Rabbit IgG (H + L)-HRP, Biorad) dilution (1:3,000) or Goat Anti-Mouse IgG (H+L)-HRP, Biorad) dilution (1:2,000). After washing, antibodies were detected by chemiluminescence using the West Pico PLUS Chemiluminescenc-Substrat (Thermo Fisher Scientific, Eugene, OR, USA).

Quantitative RT-qPCR. RNA isolation and cDNA synthesis: Messenger RNA (mRNA) of prefrontal murine cortices was isolated using RNeasy Mini Kit (Qiagen, Hilden, Germany) according to manufacturer′s instructions. Reverse transcription was performed with the ProtoScript First Strand Synthesis Kit (New England Biolabs, Frankfurt am Main, Germany) using 1 µg total RNA as template.

RT2 Profiler™ PCR array: Gene expression of Prostaglandin D2 synthase (Ptgds) and Thromboxane A Synthase 1 (Tbxas1) was analysed using a RT2 Profiler™ PCR array. Primers were synthesized by the manufacturer Qiagen and are adsorbed on the bottom of each well in a 96-well microplate, one primer pair per well. Each PCR array plate includes three housekeeping genes (Actb, Gapdh, B2m) as well as controls for genomic DNA contamination, reverse transcription efficiency and general PCR performance. Thermal cycling and fluorescence detection were performed using the CFX96 Touch Real-Time PCR Detection System from Bio-Rad Laboratories GmbH (Feldkirchen, Germany). The utilized temperature protocol includes an initial melting for 10 min at 95°C, 40 cycles of amplification (15 s at 95°C, 1 min at 60°C) followed by a melt curve. Relative gene expression was calculated using the ΔΔCt method (2-ΔΔCt). All Ct values of target cDNAs were normalized to the average of three housekeeping genes.
