# Supplementary material for: Plant Sterol-Poor Diet Is Associated with Pro-Inflammatory Lipid Mediators in the Murine Brain
Source: Int J Mol Sci. 2021 Dec 8;22(24):13207. doi: 10.3390/ijms222413207 (PMC8707069; doi:10.3390/ijms222413207)
Supplement: Supplementary file 1 [file ijms-22-13207-s001.zip › Figure S1 HF+SC sterols.pptx]

## Slide 1
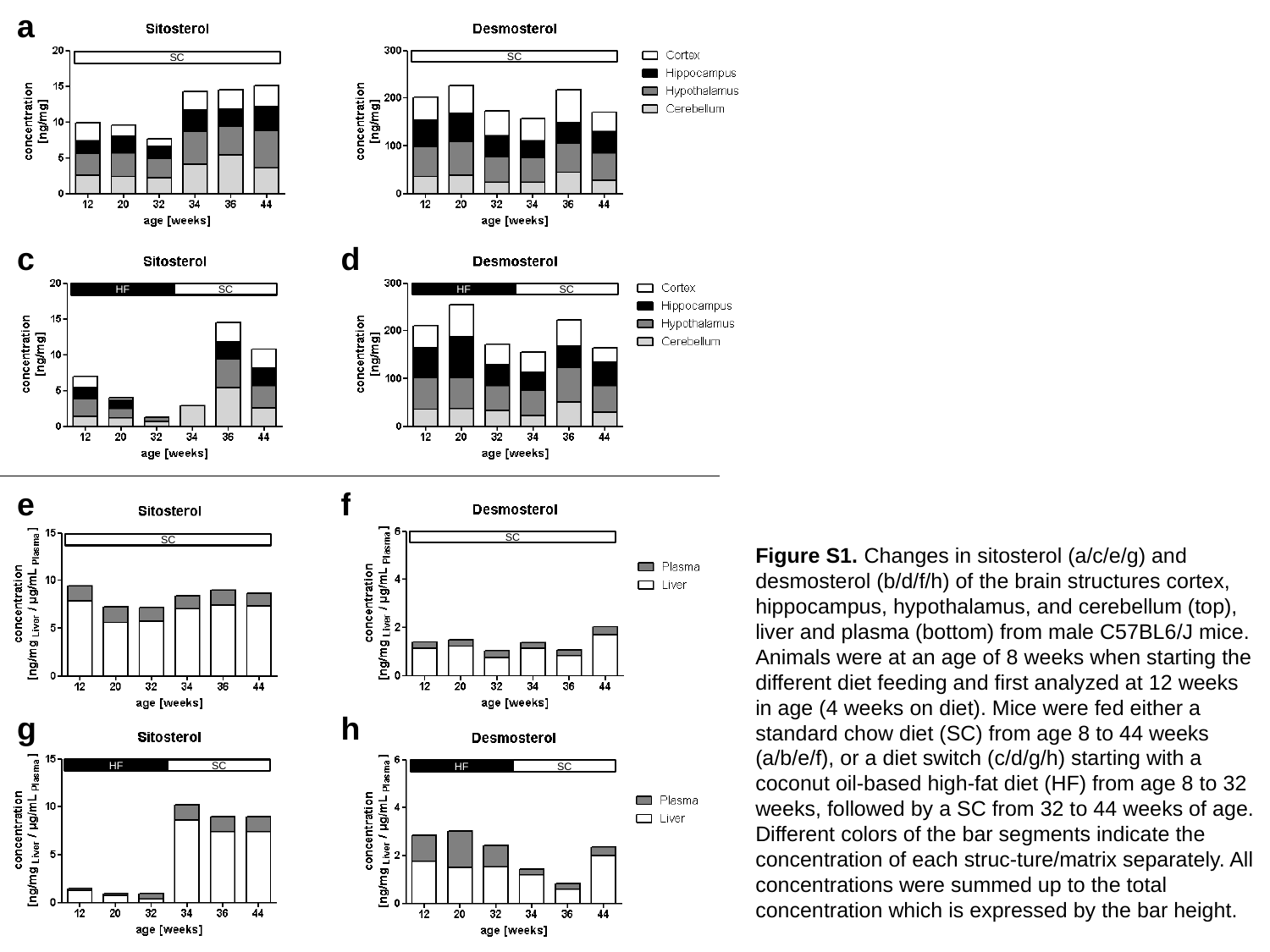

a
b
SC
SC
c
d
HF
SC
HF
SC
e
f
SC
SC
Figure S1. Changes in sitosterol (a/c/e/g) and desmosterol (b/d/f/h) of the brain structures cortex, hippocampus, hypothalamus, and cerebellum (top), liver and plasma (bottom) from male C57BL6/J mice. Animals were at an age of 8 weeks when starting the different diet feeding and first analyzed at 12 weeks in age (4 weeks on diet). Mice were fed either a standard chow diet (SC) from age 8 to 44 weeks (a/b/e/f), or a diet switch (c/d/g/h) starting with a coconut oil-based high-fat diet (HF) from age 8 to 32 weeks, followed by a SC from 32 to 44 weeks of age. Different colors of the bar segments indicate the concentration of each struc-ture/matrix separately. All concentrations were summed up to the total concentration which is expressed by the bar height.
g
h
HF
SC
HF
SC
