# Supplementary material for: Plant Sterol-Poor Diet Is Associated with Pro-Inflammatory Lipid Mediators in the Murine Brain
Source: Int J Mol Sci. 2021 Dec 8;22(24):13207. doi: 10.3390/ijms222413207 (PMC8707069; doi:10.3390/ijms222413207)
Supplement: Supplementary file 1 [file ijms-22-13207-s001.zip › Figure S10 experimental design.pptx]

## Slide 1
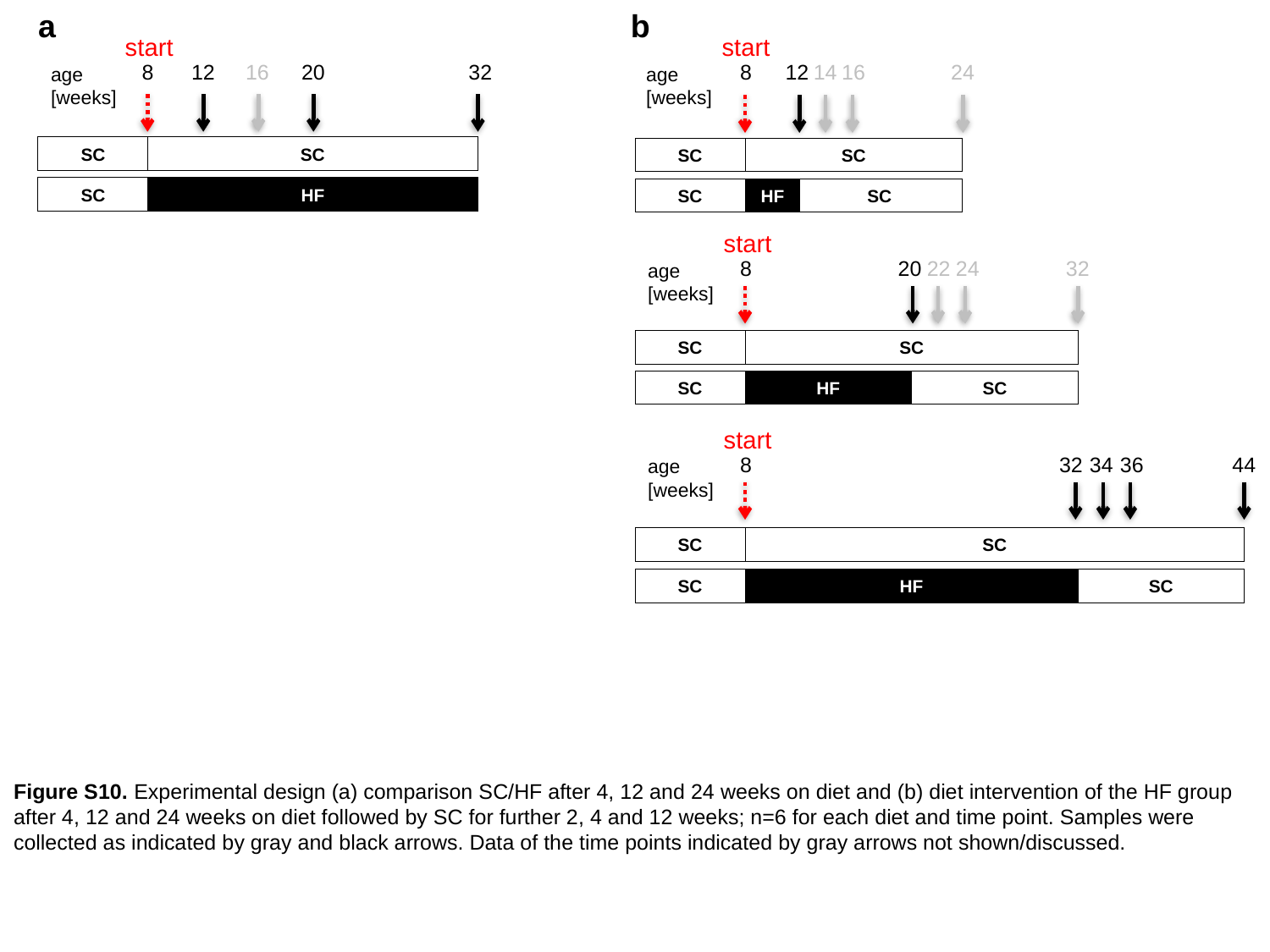

a
b
start
start
8
12
16
20
32
8
12
14
16
24
age
[weeks]
age
[weeks]
SC
SC
SC
SC
SC
HF
SC
SC
HF
start
8
20
22
24
32
age
[weeks]
SC
SC
HF
SC
SC
start
8
32
34
36
44
age
[weeks]
SC
SC
SC
SC
HF
Figure S10. Experimental design (a) comparison SC/HF after 4, 12 and 24 weeks on diet and (b) diet intervention of the HF group after 4, 12 and 24 weeks on diet followed by SC for further 2, 4 and 12 weeks; n=6 for each diet and time point. Samples were collected as indicated by gray and black arrows. Data of the time points indicated by gray arrows not shown/discussed.
