# Supplementary material for: Plant Sterol-Poor Diet Is Associated with Pro-Inflammatory Lipid Mediators in the Murine Brain
Source: Int J Mol Sci. 2021 Dec 8;22(24):13207. doi: 10.3390/ijms222413207 (PMC8707069; doi:10.3390/ijms222413207)
Supplement: Supplementary file 1 [file ijms-22-13207-s001.zip › Figure S2 body weight.pptx]

## Slide 1
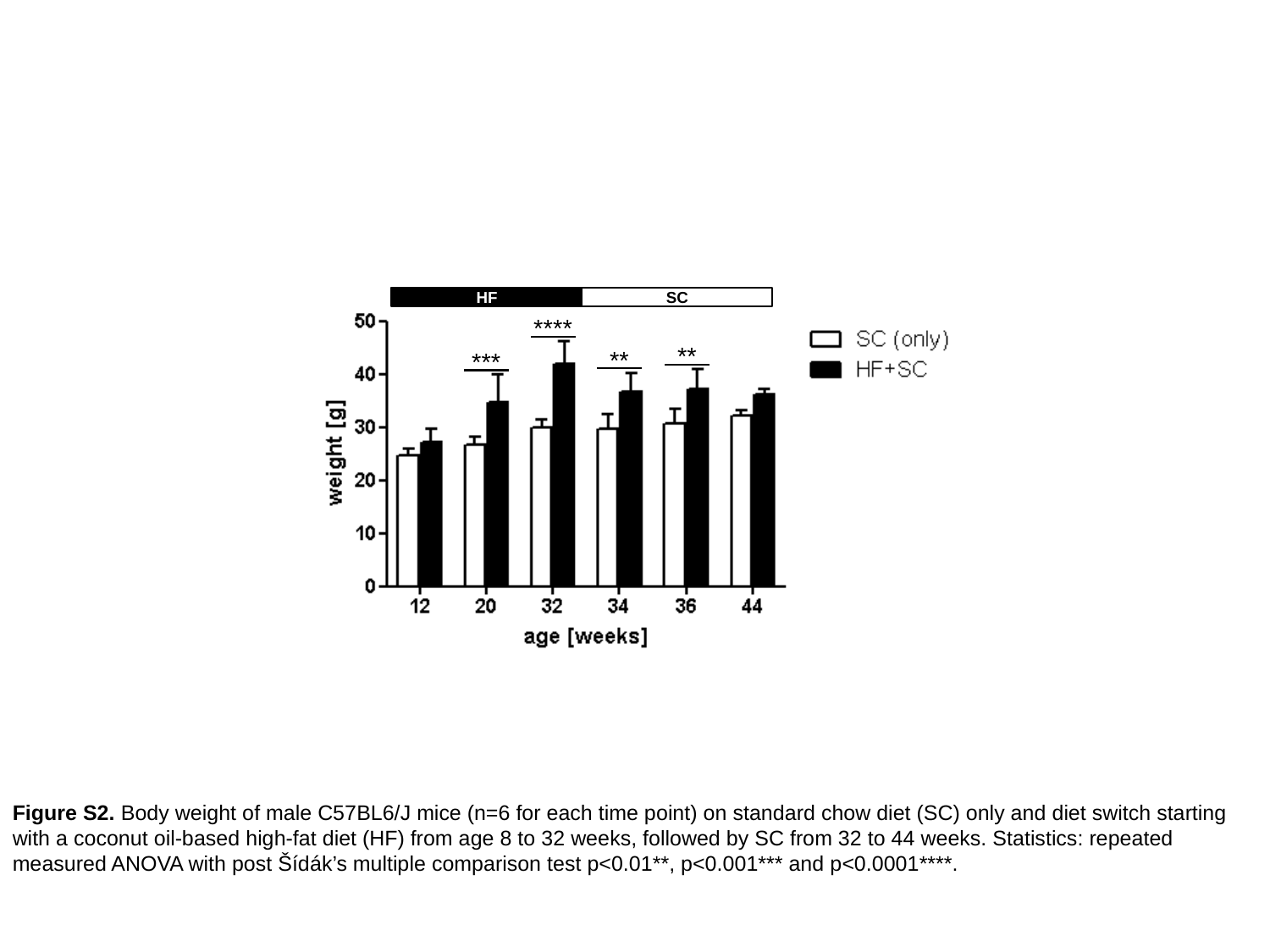

HF
SC
****
**
**
***
Figure S2. Body weight of male C57BL6/J mice (n=6 for each time point) on standard chow diet (SC) only and diet switch starting with a coconut oil-based high-fat diet (HF) from age 8 to 32 weeks, followed by SC from 32 to 44 weeks. Statistics: repeated measured ANOVA with post Šídák’s multiple comparison test p<0.01**, p<0.001*** and p<0.0001****.
