# Supplementary material for: Plant Sterol-Poor Diet Is Associated with Pro-Inflammatory Lipid Mediators in the Murine Brain
Source: Int J Mol Sci. 2021 Dec 8;22(24):13207. doi: 10.3390/ijms222413207 (PMC8707069; doi:10.3390/ijms222413207)
Supplement: Supplementary file 1 [file ijms-22-13207-s001.zip › Figure S3 FC sterols.pptx]

## Slide 1
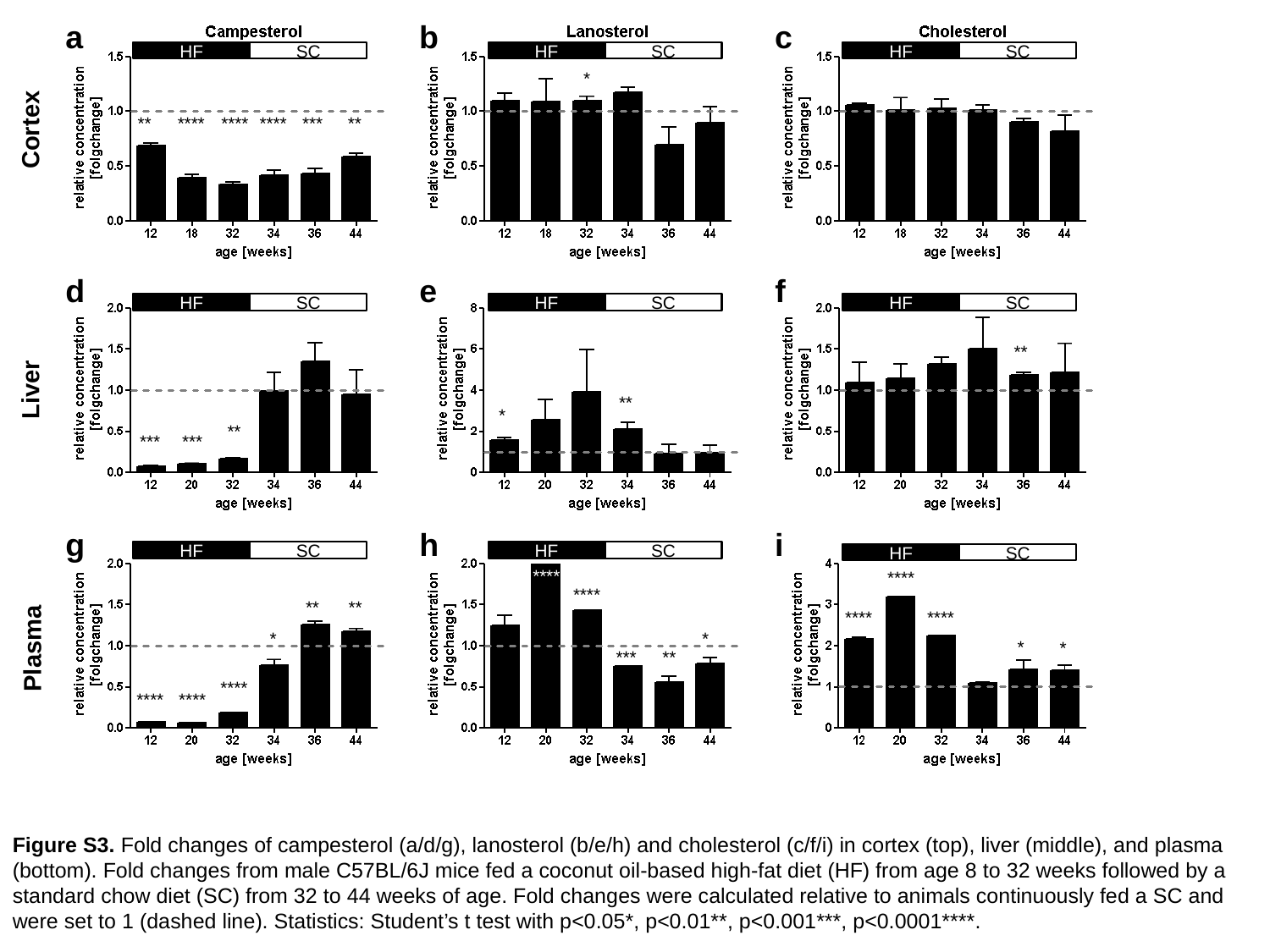

a
b
c
HF
SC
HF
SC
HF
SC
*
**
****
****
****
***
**
Cortex
d
e
f
HF
SC
HF
SC
HF
SC
**
Liver
**
*
**
***
***
g
h
i
HF
SC
HF
SC
HF
SC
****
****
****
**
**
****
****
*
*
Plasma
*
*
**
***
****
****
****
Figure S3. Fold changes of campesterol (a/d/g), lanosterol (b/e/h) and cholesterol (c/f/i) in cortex (top), liver (middle), and plasma (bottom). Fold changes from male C57BL/6J mice fed a coconut oil-based high-fat diet (HF) from age 8 to 32 weeks followed by a standard chow diet (SC) from 32 to 44 weeks of age. Fold changes were calculated relative to animals continuously fed a SC and were set to 1 (dashed line). Statistics: Student’s t test with p<0.05*, p<0.01**, p<0.001***, p<0.0001****.
