# Supplementary material for: Plant Sterol-Poor Diet Is Associated with Pro-Inflammatory Lipid Mediators in the Murine Brain
Source: Int J Mol Sci. 2021 Dec 8;22(24):13207. doi: 10.3390/ijms222413207 (PMC8707069; doi:10.3390/ijms222413207)
Supplement: Supplementary file 1 [file ijms-22-13207-s001.zip › Figure S4 obob sterols+eicos_brain_liver.pptx]

## Slide 1
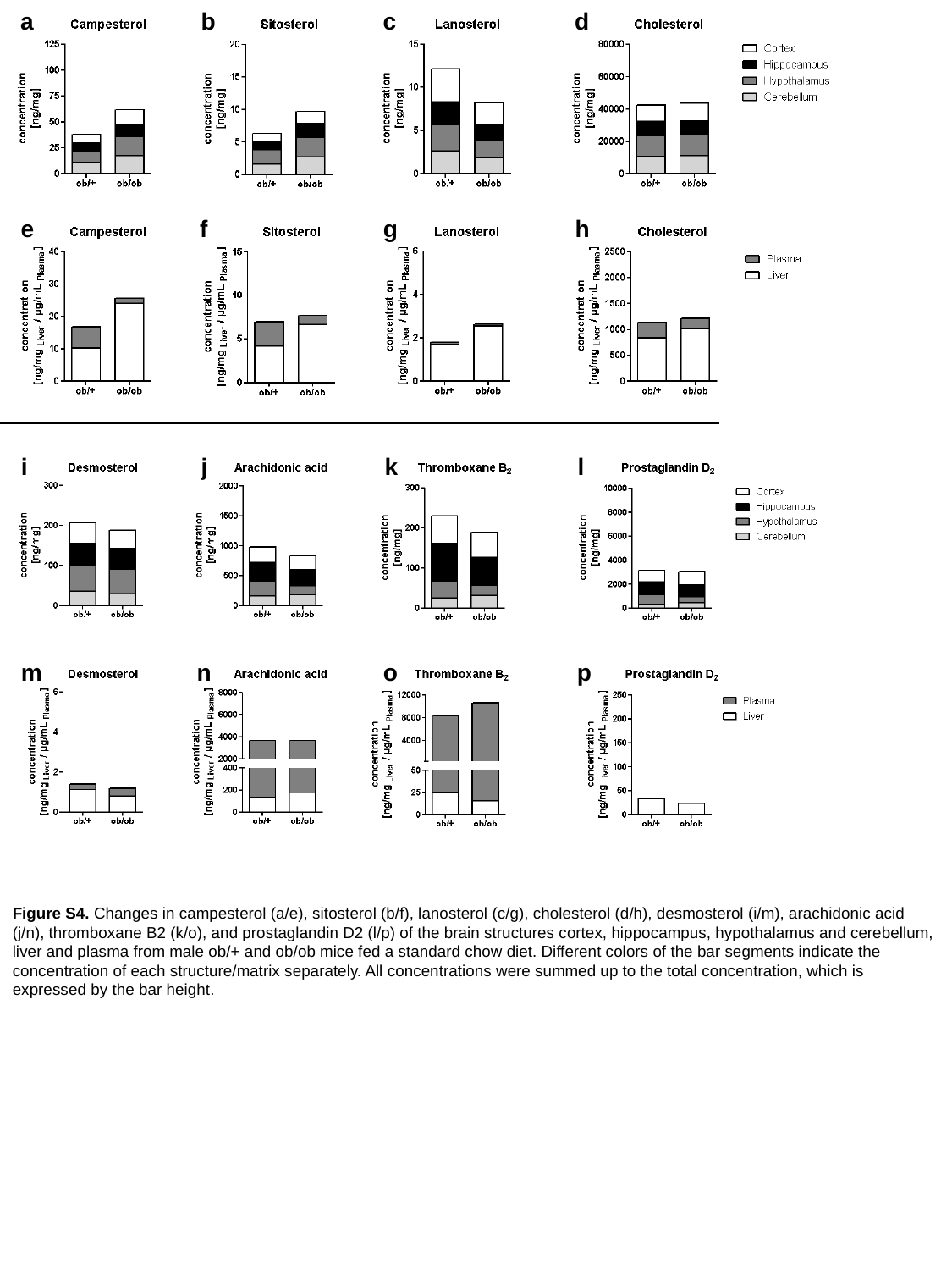

a
b
c
d
e
f
g
h
i
j
k
l
m
n
o
p
Figure S4. Changes in campesterol (a/e), sitosterol (b/f), lanosterol (c/g), cholesterol (d/h), desmosterol (i/m), arachidonic acid (j/n), thromboxane B2 (k/o), and prostaglandin D2 (l/p) of the brain structures cortex, hippocampus, hypothalamus and cerebellum, liver and plasma from male ob/+ and ob/ob mice fed a standard chow diet. Different colors of the bar segments indicate the concentration of each structure/matrix separately. All concentrations were summed up to the total concentration, which is expressed by the bar height.
