# Supplementary material for: Plant Sterol-Poor Diet Is Associated with Pro-Inflammatory Lipid Mediators in the Murine Brain
Source: Int J Mol Sci. 2021 Dec 8;22(24):13207. doi: 10.3390/ijms222413207 (PMC8707069; doi:10.3390/ijms222413207)
Supplement: Supplementary file 1 [file ijms-22-13207-s001.zip › Figure S5 SC+HF eicos.pptx]

## Slide 1
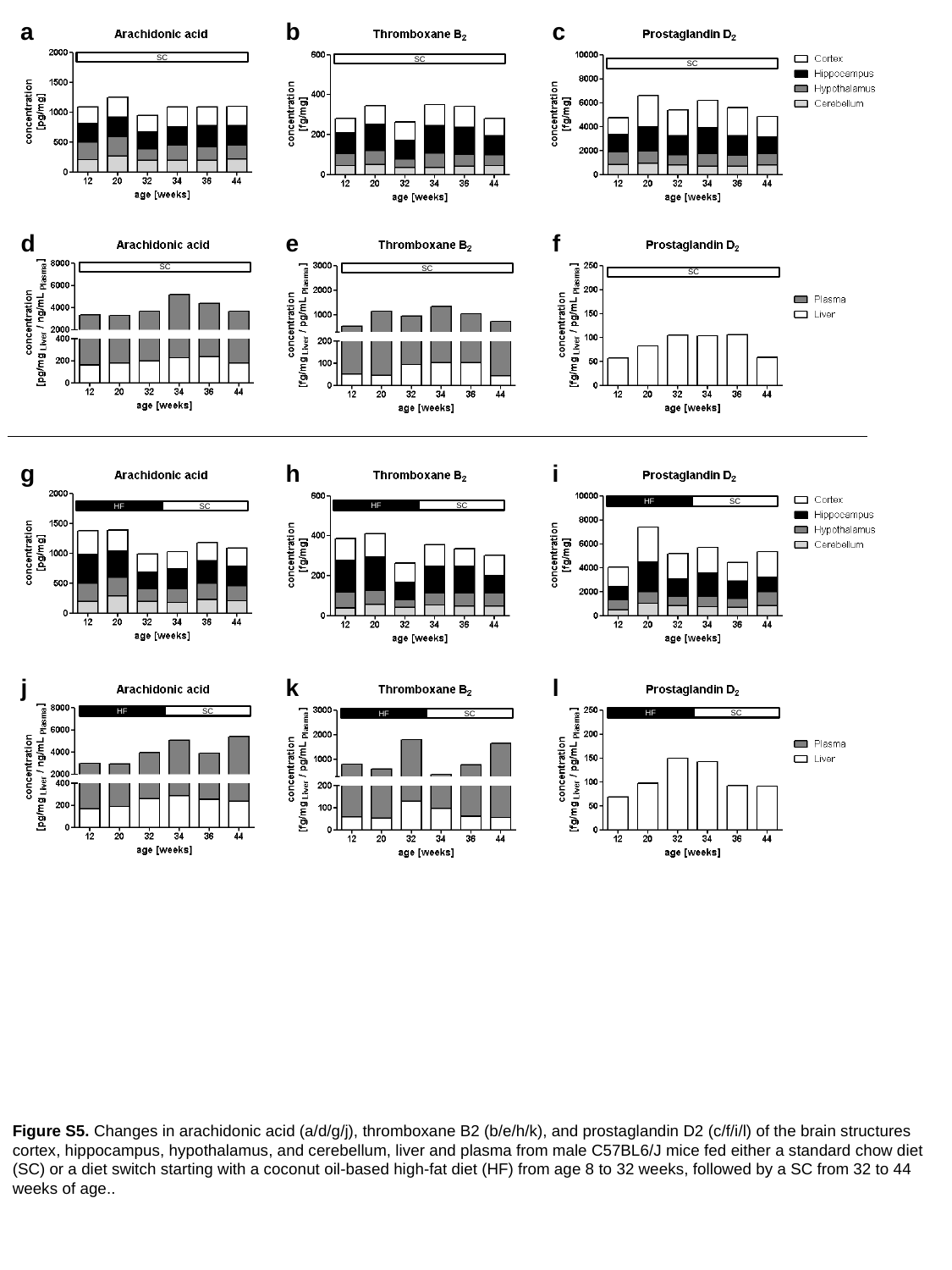

a
b
c
SC
SC
SC
d
e
f
SC
SC
SC
g
h
i
HF
SC
HF
SC
HF
SC
j
k
l
HF
SC
HF
SC
HF
SC
Figure S5. Changes in arachidonic acid (a/d/g/j), thromboxane B2 (b/e/h/k), and prostaglandin D2 (c/f/i/l) of the brain structures cortex, hippocampus, hypothalamus, and cerebellum, liver and plasma from male C57BL6/J mice fed either a standard chow diet (SC) or a diet switch starting with a coconut oil-based high-fat diet (HF) from age 8 to 32 weeks, followed by a SC from 32 to 44 weeks of age..
