# Supplementary material for: Plant Sterol-Poor Diet Is Associated with Pro-Inflammatory Lipid Mediators in the Murine Brain
Source: Int J Mol Sci. 2021 Dec 8;22(24):13207. doi: 10.3390/ijms222413207 (PMC8707069; doi:10.3390/ijms222413207)
Supplement: Supplementary file 1 [file ijms-22-13207-s001.zip › Figure S6 correlation CA+SI-PGD2_TxB2_SC.pptx]

## Slide 1
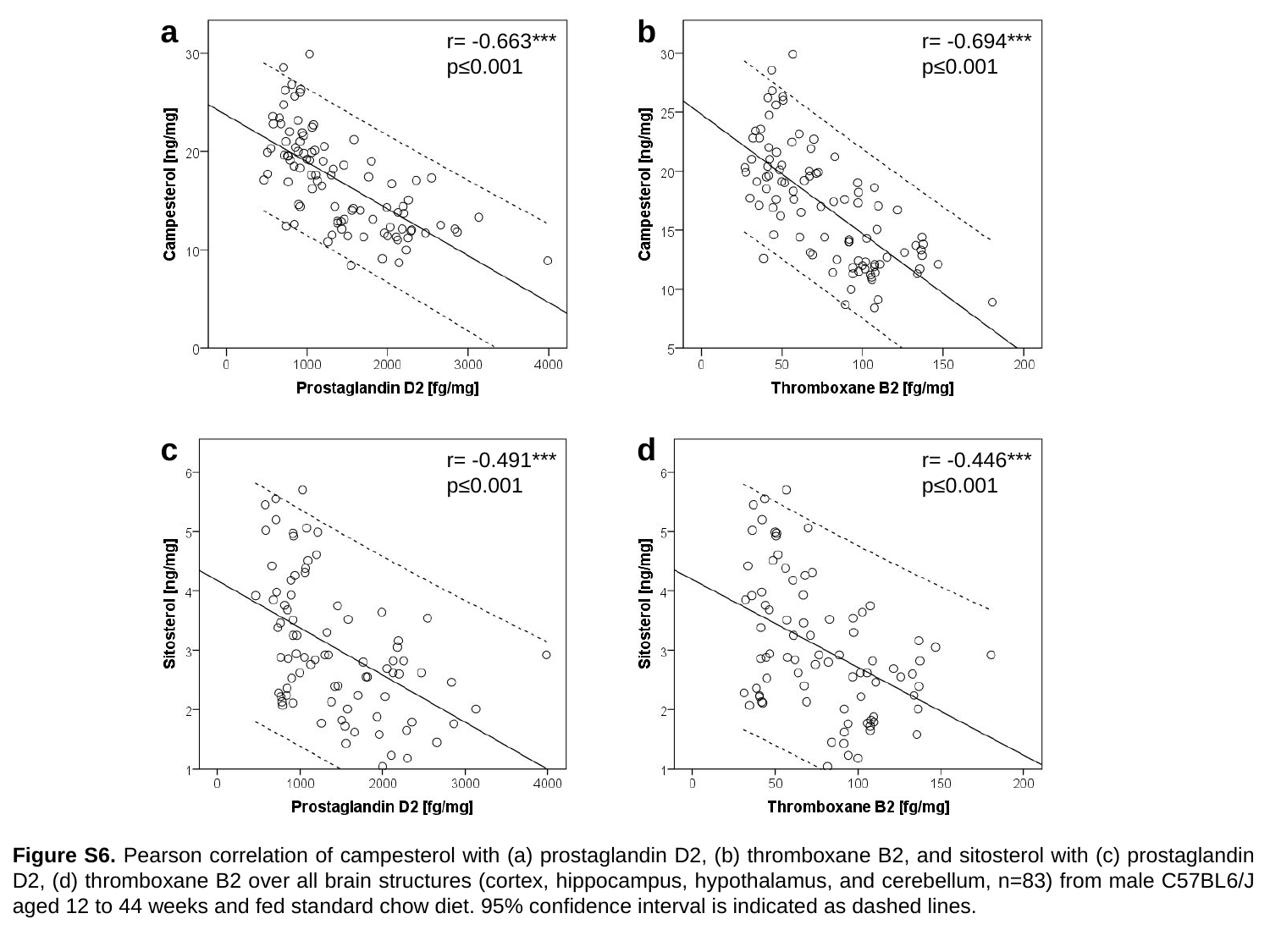

a
b
r= -0.663***
p≤0.001
r= -0.694***
p≤0.001
c
d
r= -0.491***
p≤0.001
r= -0.446***
p≤0.001
Figure S6. Pearson correlation of campesterol with (a) prostaglandin D2, (b) thromboxane B2, and sitosterol with (c) prostaglandin D2, (d) thromboxane B2 over all brain structures (cortex, hippocampus, hypothalamus, and cerebellum, n=83) from male C57BL6/J aged 12 to 44 weeks and fed standard chow diet. 95% confidence interval is indicated as dashed lines.
