# Supplementary material for: Plant Sterol-Poor Diet Is Associated with Pro-Inflammatory Lipid Mediators in the Murine Brain
Source: Int J Mol Sci. 2021 Dec 8;22(24):13207. doi: 10.3390/ijms222413207 (PMC8707069; doi:10.3390/ijms222413207)
Supplement: Supplementary file 1 [file ijms-22-13207-s001.zip › Figure S7 Ptgds-Txbas1 4-24W.pptx]

## Slide 1
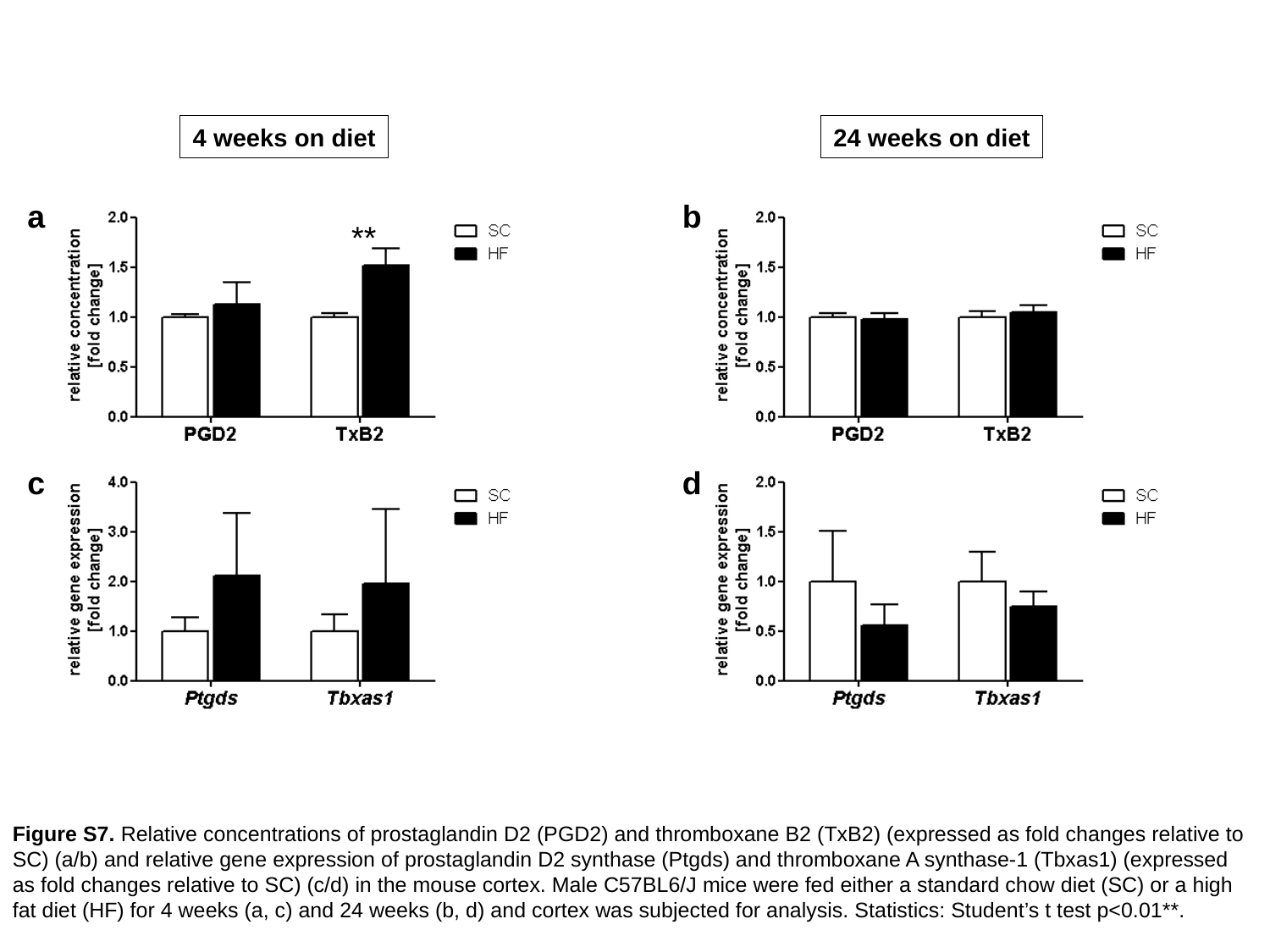

4 weeks on diet
24 weeks on diet
a
b
**
c
d
Figure S7. Relative concentrations of prostaglandin D2 (PGD2) and thromboxane B2 (TxB2) (expressed as fold changes relative to SC) (a/b) and relative gene expression of prostaglandin D2 synthase (Ptgds) and thromboxane A synthase-1 (Tbxas1) (expressed as fold changes relative to SC) (c/d) in the mouse cortex. Male C57BL6/J mice were fed either a standard chow diet (SC) or a high fat diet (HF) for 4 weeks (a, c) and 24 weeks (b, d) and cortex was subjected for analysis. Statistics: Student’s t test p<0.01**.
