# Supplementary material for: Plant Sterol-Poor Diet Is Associated with Pro-Inflammatory Lipid Mediators in the Murine Brain
Source: Int J Mol Sci. 2021 Dec 8;22(24):13207. doi: 10.3390/ijms222413207 (PMC8707069; doi:10.3390/ijms222413207)
Supplement: Supplementary file 1 [file ijms-22-13207-s001.zip › Figure S8 Western blot lipid rafts.pptx]

## Slide 1
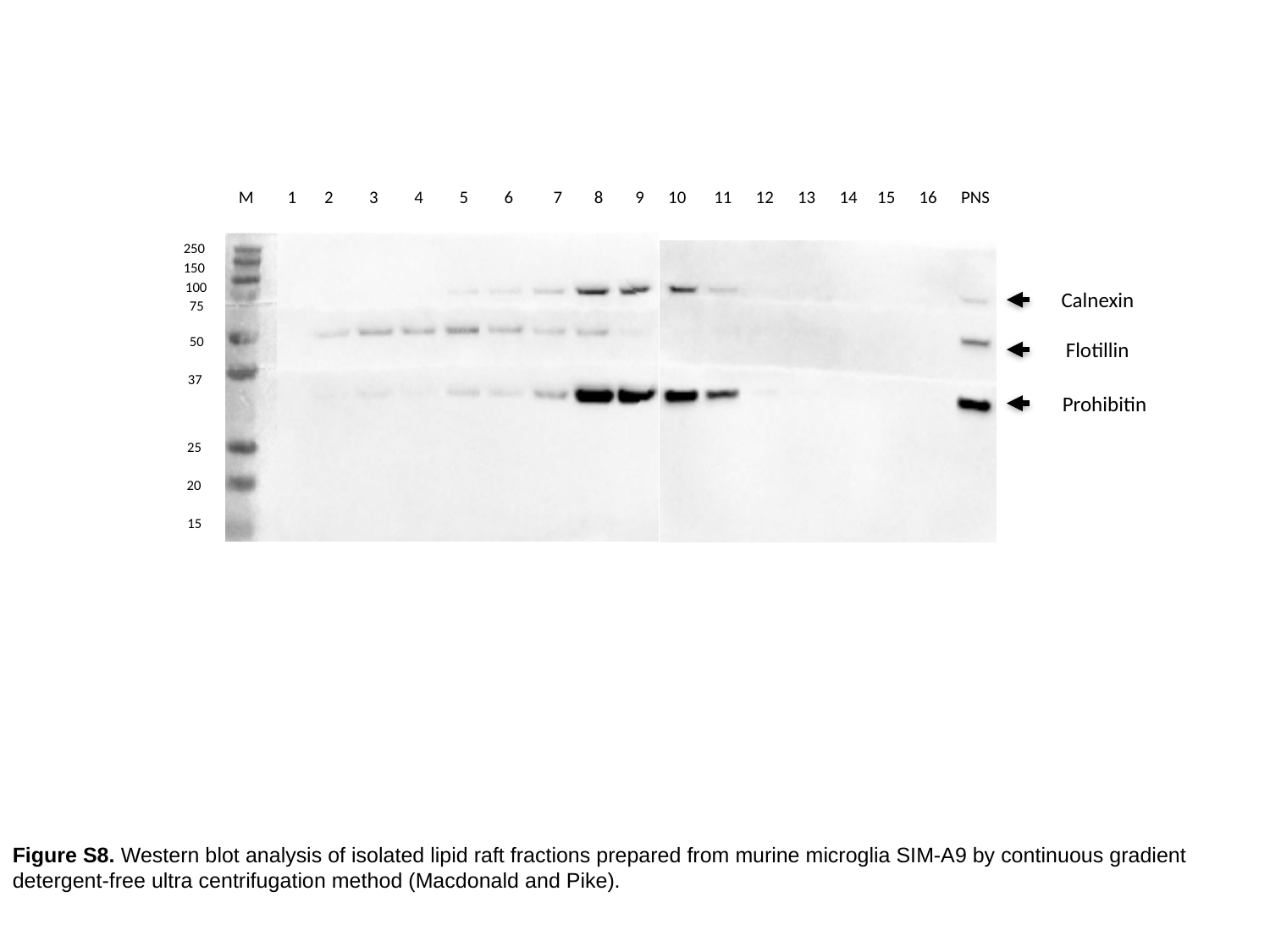

1 2 3 4 5 6 7 8 9 10 11 12 13 14 15 16 PNS
M
250
150
100
75
50
37
25
20
15
Calnexin
Flotillin
Prohibitin
Figure S8. Western blot analysis of isolated lipid raft fractions prepared from murine microglia SIM-A9 by continuous gradient detergent-free ultra centrifugation method (Macdonald and Pike).
