# Supplementary material for: Plant Sterol-Poor Diet Is Associated with Pro-Inflammatory Lipid Mediators in the Murine Brain
Source: Int J Mol Sci. 2021 Dec 8;22(24):13207. doi: 10.3390/ijms222413207 (PMC8707069; doi:10.3390/ijms222413207)
Supplement: Supplementary file 1 [file ijms-22-13207-s001.zip › Figure S9 Microglia Aktivierung PS.pptx]

## Slide 1
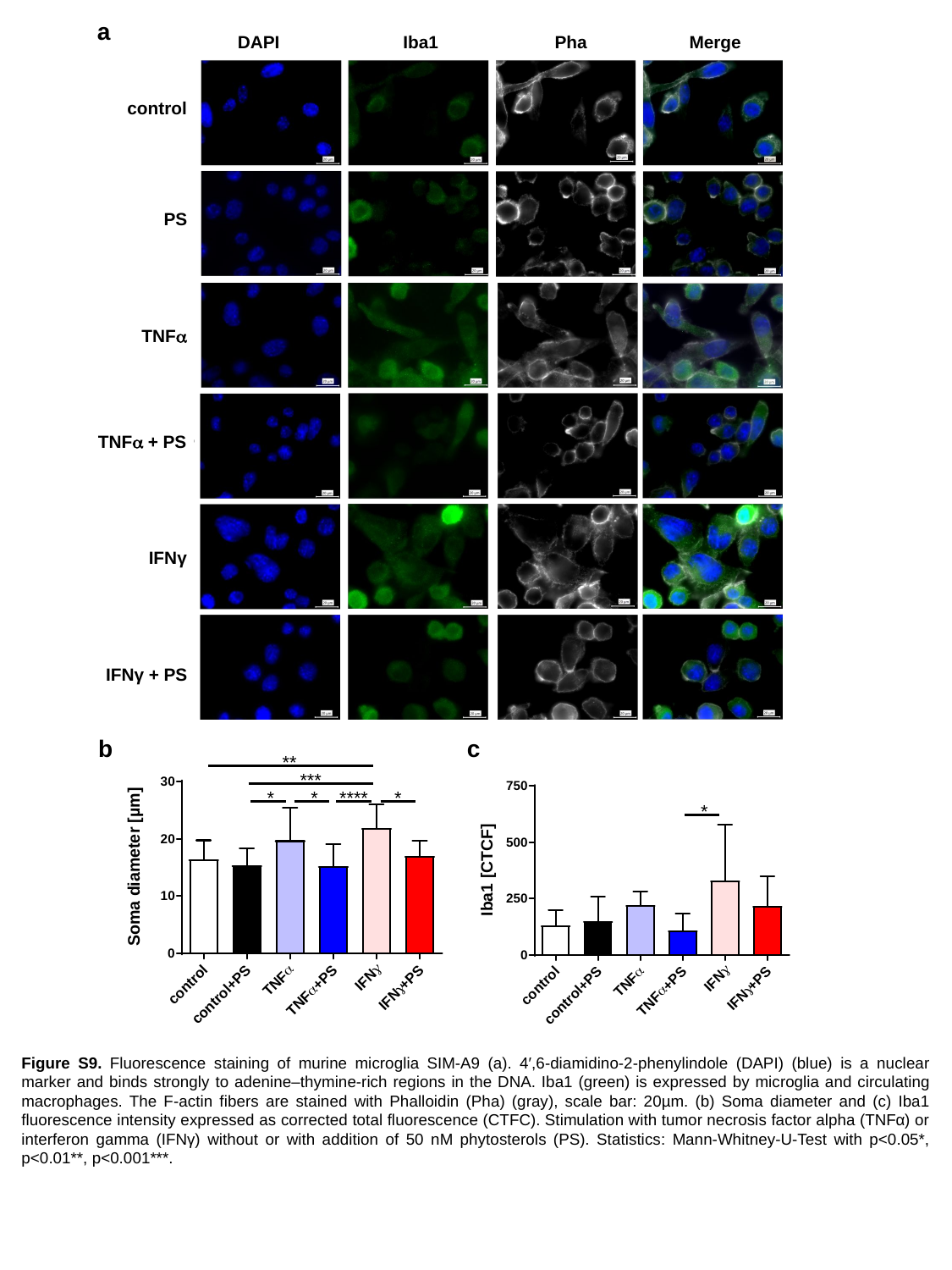

a
DAPI
Iba1
Pha
Merge
control
PS
TNFα
TNFα + PS
IFNγ
IFNγ + PS
b
**
***
*
*
****
*
c
*
Figure S9. Fluorescence staining of murine microglia SIM-A9 (a). 4′,6-diamidino-2-phenylindole (DAPI) (blue) is a nuclear marker and binds strongly to adenine–thymine-rich regions in the DNA. Iba1 (green) is expressed by microglia and circulating macrophages. The F-actin fibers are stained with Phalloidin (Pha) (gray), scale bar: 20µm. (b) Soma diameter and (c) Iba1 fluorescence intensity expressed as corrected total fluorescence (CTFC). Stimulation with tumor necrosis factor alpha (TNFα) or interferon gamma (IFNγ) without or with addition of 50 nM phytosterols (PS). Statistics: Mann-Whitney-U-Test with p<0.05*, p<0.01**, p<0.001***.
